# Near-Infrared Responsive Composites of Poly-3,4-Ethylenedioxythiophene with Fullerene Derivatives

**DOI:** 10.3390/polym17010014

**Published:** 2024-12-25

**Authors:** Oxana Gribkova, Varvara Kabanova, Ildar Sayarov, Alexander Nekrasov, Alexey Tameev

**Affiliations:** A.N. Frumkin Institute of Physical Chemistry and Electrochemistry RAS, Leninskii Prospect 31, Moscow 119071, Russia; gribkova@elchem.ac.ru (O.G.); kabanovavar@phyche.ac.ru (V.K.); sayarovir@phyche.ac.ru (I.S.); secp@elchem.ac.ru (A.N.)

**Keywords:** PEDOT, fullerene, electrochemical polymerization, spectroelectrochemistry, photoconductivity

## Abstract

Electrochemical polymerization of 3,4-ethylenedioxythiophene in the presence of water-soluble fullerene derivatives was investigated. The electronic structure, morphology, spectroelectrochemical, electrochemical properties and near-IR photoconductivity of composite films of poly(3,4-ethylenedioxythiophene) with fullerenes were studied for the first time. It was shown that fullerene with hydroxyl groups creates favorable conditions for the formation of PEDOT chains and more effectively compensates for the positive charges on the PEDOT chains. The near-IR photoconductivity results from the generation of charge carriers due to electron transfer from the photoexcited PEDOT molecule to the fullerene acceptor.

## 1. Introduction

Electrochemical synthesis of conductive polymers combines the polymerization and deposition of polymer films of the desired thickness onin a single step. The thickness of the polymer films can be easily adjusted by controlling the total amount of charge spent on the electropolymerization. The electrochemically deposited layers usually are more uniform and the electrical contact between the electrode and layer is stronger than that of the layer cast from a solution. By comparison, the very popular poly(3,4-ethylenedioxythiophene):polystyrenesulfonate (PEDOT:PSS) layers of typical thickness 20 to 50 nm obtained by spin-coating tend to form relatively large grains and have a high defect density [1], which results in unfavorable reverse recombination of charge carriers and, consequently, high noise in the current, which is detrimental to the performance of organic photodiodes [2].

Composite layers of conductive polymers can be obtained by introducing various carbon nanomaterials into the polymerization solution. PEDOT has a narrow band gap, which ensures reversible electrochemical p- and n-doping, excellent thermal and electrochemical stability, and the possibility of using environmentally friendly aqueous electrolytes without polymer degradation. The formation of PEDOT composites with fullerenes can lead to products with combined properties inherent in both components. PEDOT can act as an electron donor that absorbs light in a wide range of the spectrum, while fullerene is a well-known electron acceptor that can facilitate the separation of photogenerated electron–hole pairs. The combination of both compounds in one thin film allows for obtaining a material with unique properties. Electrically conductive PEDOT in semi-oxidized and oxidized states has electron levels of polarons and bipolarons that are located inside the band gap and provide absorption of the polymer in the near-IR range. Thus, the introduction of fullerene derivatives into PEDOT can promote photoconductivity in the near-IR range of the spectrum.

There are a small number of works in the literature describing the electrochemical synthesis of composite layers of PEDOT with fullerenes. Thus, to obtain functional layers in organic solar cells (OSC), the electrocodeposition of thiophene and C60 [3] or thiophene derivative and C60 [4] were carried out in a wide range of potential cycling in organic solvents. In another case, monomers of 3,4-ethylenedioxythiophene (EDOT) containing pendant fragments C60 were synthesized and then were electropolymerized on an electrode [5,6]. To obtain electrochromic layers, C60 was used as a doping anion to compensate for the positive charge of the oxidized polymer [7,8]. As a result, C60 was deposited on the film simultaneously with electropolymerized EDOT or its derivatives and was evenly distributed within the layer. In [9], photoactive hybrid PEDOT films with C60 and C60 with a grafted thiophene anchor group were electrochemically synthesized and their photocurrent generation properties in the presence of methyl viologen in the range of 400–700 nm were investigated. In all described cases, electropolymerization was carried out in organic solvents with background electrolytes. In [10] only, PEDOT films were electrochemically deposited on electrodes using aqueous solutions containing EDOT and fullerene sulfonated calixarenes.

In cases of using PEDOT–fullerene as a photogeneration layer in a diode structure, it should be isolated from the anode by a hole-transport (electron-blocking) layer. A commercial PEDOT–PSS composition is most often used as such a layer. In order to create an OSC, the authors of [3,4] performed a copolymerization of thiophene and C60 on the layer of electrodeposited PEDOT. To improve film formation and interfacial charge transfer between the polymer film and the ITO electrode, the authors of [11] used an electrodeposited PEDOT–polystyrene sulfonic acid (PSSA) sublayer. Our previous studies [12,13] demonstrated the efficiency of electrodeposition of composites of PEDOT with Zn and Cu phthalocyaninates and polypyrrole with Zn phthalocyaninate on a thin sublayer of electrode deposited PEDOT complex with poly(2-acrylamido-2-methyl-1-propanesulfonic acid) (PAMPSA). This sublayer was chosen based on our studies of the effect of the hole-transport layer composition on the performance of inverted perovskite solar cells (PSCs) [14]. A PSC with a PEDOT–PAMPSA layer demonstrated the maximum short-circuit current and efficiency. Earlier [15], we prepared electrodeposited PEDOT–fullerenol photosensitive layers and demonstrated their applicability for the development of organic photodiodes. In this case PEDOT-fullerenol layers were electrodeposited without sublayer. When studying the influence of the structure of fullerene derivatives on the electrodeposition of PEDOT, it was found that in the presence of fullerene with 5 carboxyl groups PEDOT can only be electrodeposited onto thin PEDOT sublayer.

In the presented work, we have carried out a comparative investigation of the electrochemical synthesis of PEDOT composite films with water-soluble fullerenes of various structures on the thin sublayer of electrodeposited PEDOT–PAMPSA. The electronic structure and morphology, and the spectroelectrochemical and electrochemical properties of PEDOT composite films with fullerene derivatives were studied. Finally, the performance of the obtained layers in near-IR organic photodiodes was compared.

## 2. Materials and Methods

### 2.1. Materials

**Figure 1:**
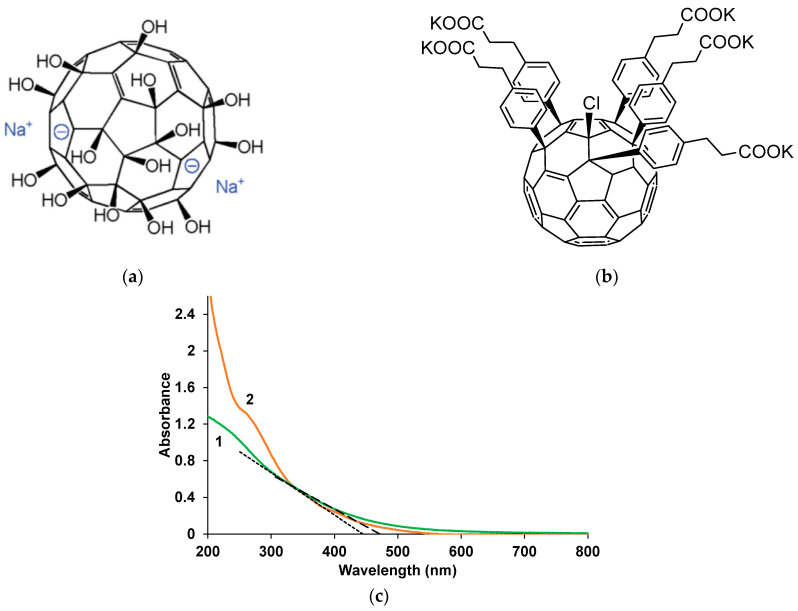
Structure (**a**,**b**) [16] and electronic absorption spectra (**c**) of 0.0001 M aqueous solution of NaFl (1) and KPCF (2).

Na^+^-containing fullerene with hydroxyl groups (Na_4_[C_60_(OH)_x_], where x~30) (NaFl) [16], synthesized according to the method described in [17] and K^+^-containing fullerene with 5 carboxyl groups (KPCF) [16], synthesized according to the method described in [18], were used. Figure 1 shows the structural formulas (a, b) and spectra of aqueous solutions (c) of the fullerenes used. It is evident that they absorb in the UV–visible regions up to 600 nm. By drawing tangents to the decay of the absorption bands of fullerenes, we determined the widths of their optical band gaps (Table 2). The shoulder near 260–280 nm, observed for KPCF, corresponds to absorption of phenyl groups.

In NaFl, the most probable localization of the negative charge is on the oxygen atoms with partial localization on the carbon framework [16,19]. Amphiphilic water-soluble fullerene derivative KPCF tends to form various supramolecular structures including vesicles and their clusters in aqueous media. According to dynamic light scattering data, the hydrodynamic radii of the vesicles are 20, 70 nm and more than 10 μm [20].

To reveal the influence of these aggregates on the ability of the substituted fullerenes to act as charge-compensating counter ions during EDOT electropolymerization, we have measured the zeta-potentials in 0.002 M solutions of NaFl or KPCF with the addition of 0.001 M NaCl. The results are given in the Supplementary Materials (SM). From Figure S1a, it is clear that NaFl exhibits one sharp peak, corresponding to the potential values near −24 mV. On the contrary, KPCF (Figure S1b) exhibits one sharp and one wide peak, the first corresponding to the potential values near −12–15 mV and the second one to those near −39–42 mV. The sharp peaks most possibly correspond to non-associated fullerene derivatives. The fact that zeta-potential of single NaFl is higher than that of single KPCF may be explained by higher content of charged moieties on the surface of the former fullerene. The wide peak for KPCF most possibly corresponds to the abovementioned aggregates. By comparing potentials of sharp and wide peaks, one may suppose that the aggregates include approximately three molecules of KPCF on the average. However, the long tail of the wide peak extending to the area with more negative potentials testifies to the existence of aggregates of a higher degree association. Taking into account that the KPCF molecule is asymmetric and has hydrophilic (-COOK groups) and hydrophobic (fullerene body) parts, the most probable structure of the aggregates in aqueous medium is fullerene bodies in the inner sphere surrounded by -COOK groups in the outer sphere.

### 2.2. PEDOT Synthesis in the Presence of Fullerenes

EDOT (Sigma–Aldrich (St. Louis, MO, USA)) was distilled under argon. The freshly distilled product was used. Fullerenes were dissolved in water to the desired concentration, then EDOT was added and the solution was intensively stirred for 2 h with heating to ~60 °C.

The PEDOT–PAMPSA sublayer was electrodeposited onto glass–FTO electrodes (7 Ohm/sq., Solaronix SA, Aubonne, Switzerland) with an area of ~1.3 CM^2^ in galvanostatic mode at a current density of 0.05 mA/cm^2^ in an aqueous solution containing 0.01 M EDOT and 0.02 M PAMPSA (Sigma–Aldrich, 15% aqueous solution, Mw ≈ 2·10^6^). Electrodeposition of the sublayer was carried out until the electropolymerization charge of 7 mC/cm^2^ was reached, which corresponded to a thickness of ~30 nm.The thickness was measured with a stylus profiler Alfa-Step D-100 (KLA Corp., Milpitas, CA, USA).

For the polymerization of EDOT, the following aqueous solutions were prepared: 0.01 M EDOT and 0.0014 M NaFl; 0.01 M EDOT and 0.004 M KPCF. These optimal concentrations were chosen on the base of preliminary experiments. Electrodeposition of PEDOT composite films was carried out in potentiodynamic (PD) in the potential range of −0.6–1.0 V at a scan rate of 50 mV/s, galvanostatic (GS) at a current density of 0.05 mA/cm^2^ and potentiostatic (PS) at a potential of 0.9 V on FTO electrodes covered with the PEDOT–PAMPSA sublayer. Composite PEDOT layers were electrodeposited until a charge of 43 mC/cm^2^ was reached. The total charge (including sublayer) was 50 mC/cm^2^, and the total layer thickness was 200–300 nm. The polymerization was carried out in a three-electrode cell based on a 2 cm spectrophotometric cuvette with a special lid for fixing the electrodes and the salt bridge to a separate volume with a reference electrode. Platinum foil was used as a counter electrode, and a saturated silver–silver chloride electrode (Ag/AgCl) was used as a reference electrode. All potentials in this work are presented relative to this electrode.

To determine the lowest unoccupied molecular orbital (LUMO) and highest occupied molecular orbital (HOMO) energy levels, the composite layers of PEDOT were deposited in the GS mode on a Pt electrode with an area of ~0.5 cm^2^ with a PEDOT–PAMPSA sublayer until a charge of 100 mC/cm^2^ was reached.

To study the photoelectric properties of the composite films, electrochemical polymerization of EDOT was carried out on optically transparent glass–ITO (indium-tin oxide) electrodes (15 Ohm/sq., Kintec, Hong Kong, China) with an area of 1.12 cm^2^ with PEDOT–PAMPSA as a sublayer in the presence of NaFl and KPCF in the GS mode until a charge of 50 mC/cm^2^ was reached.

### 2.3. Preparation and Characterization Techniques

During the EDOT electropolymerization, electrochemical data and, simultaneously, in situ optical absorption spectra in the UV–visible region (350–950 nm) were recorded. Control and recording of the electrochemical parameters during the synthesis and electrochemical studies of the obtained films were carried out using an Autolab PGSTAT302N potentiostat (Metrohm, Utrecht, The Netherlands). Optical absorption spectra during the PEDOT synthesis with a repetition frequency of 2 s, as well as spectroelectrochemical studies of the obtained films at fixed potentials in an aqueous solution of 0.5 M NaClO_4_, were carried out using a high-speed scanning single-beam spectrophotometer Avantes 2048 (Avantes BV, Apeldoorn, The Netherlands).

The LUMO and HOMO energy levels were determined by cyclic voltammetry (CV) in non-aqueous medium. The detailed procedure is described in the Supplementary Materials. The Fermi level and valence band energy states of the solid films were measured by ultraviolet photoelectron spectroscopy (UPS) using a Thermo Fisher Scientific ESCAlab 250Xi spectrometer (Waltham, MA, USA), with a base pressure of 1·10^−10^ mbar. For the UPS measurements, an ultraviolet (He I, 21.22 eV) light source was employed and a sample bias of −5 V was applied to obtain the secondary electron cutoff.

The electron absorption spectra of the obtained films in air in the range of 300–1300 nm were recorded using a Shimadzu UV-3101PC spectrophotometer (Shimadzu Deutschland GmbH, Duisburg, Germany).

The ζ-potential of the fullerenes solutions was measured by means of a Zetasizer Nano ZS (Malvern, England) analyzer.

The surface morphology of the composite films obtained in the galvanostatic mode was investigated using an Enviroscope atomic force microscope (AFM) with a Nanoscope V controller (Bruker, Billerica, MA, USA) in the semi-contact mode. Scanning electron microscopy (SEM) of the obtained films was performed using a Tescan Amber GMH scanning microscope (Tescan Orsay Holding, a.s., Brno–Kohoutovice, Czech Republic). The images were obtained using an Everhart–Thornley SE detector at ×10,000–300,000 magnifications and an accelerating voltage of 0.5 kV.

The photoelectric characteristics of the composite layers were investigated in samples of the ITO/sublayer/composite/C_60_/BCP/Al diode structure, where C_60_ (MST, St. Petersburg, Russia) is a 40 nm fullerene layer as an electron acceptor and transport layer, BCP (Kintec, Hong Kong, China) is an 8 nm layer of 2,9-dimethyl-4,7-diphenyl-1,10-phenanthroline blocking holes, and Al is an electrode (80 nm) All layers, except the composite and sublayer, were deposited by vacuum evaporation.

A standard Keithley 2400 source-meter unit (Solon, OH, USA) was used to perform photoelectric measurements. The samples were illuminated with a xenon UV-IR light source, a model 66477 (Newport Corp., Irvine, CA, USA) through a combination of SZS-26 and KS19 filters (LOMO, St. Petersburg, Russia), which transmit radiation within the 700 nm and 900 nm bandwidth. The radiation power of the light flux incident on the samples was measured using a PE25-SH Ophir energy sensor (Ophir, Jerusalem, Israel). All photoelectric measurements were performed in a sealed glove box in a dry argon atmosphere.

## 3. Results and Discussion

### 3.1. Electropolymerization of EDOT in the Presence of Fullerenes

**Figure 2:**
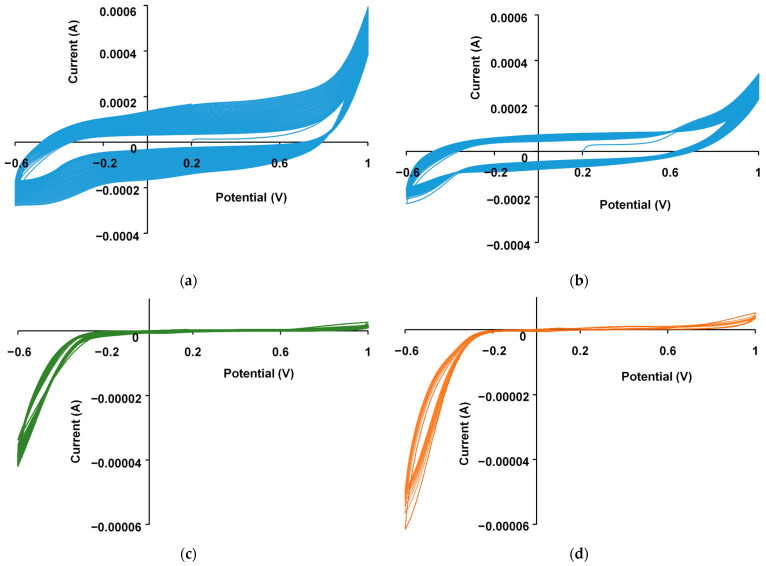
Cyclic voltammograms in aqueous solutions: (**a**) FTO electrode with a PEDOT–PAMPSA sublayer in 0.01 M EDOT and 0.0014 M NaFl; (**b**) FTO electrode with the sublayer in 0.01 M EDOT and 0.004 M KPCF; (**c**) bare FTO electrode in 0.0014 M NaFl and (**d**) bare FTO electrode in 0.004 M KPCF. The potential scan rate was 50 mV/s.

Figure 2 shows the cyclic voltammograms (CVA) obtained during the synthesis of PEDOT in the PD mode in the potential range from −0.6 to 1.0 V. It is evident that the shape of the curve is characteristic of PEDOT films obtained in aqueous solutions [17] (Figure S2). The onset potentials of EDOT oxidation in aqueous solutions of NaFl and KPCF are 0.76 and 0.57 V, respectively. It is evident that in the presence of NaFl, the synthesis of PEDOT proceeds faster with higher currents. The fullerenes used do not exhibit electroactivity in this region of potential cycling (Figure 2c,d).

**Figure 3:**
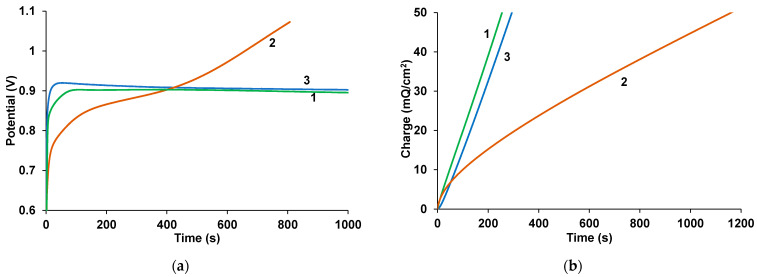
Time dependency of the potential during GS (**a**) and the charge during PS (**b**) electropolymerization of 0.01 M EDOT in the aqueous solutions of 0.0014 M NaFl (1) and 0.004 M KPCF (2) and 0.02 M PAMPSA (3).

The changes in potential over time obtained during the polymerization of EDOT in the presence of fullerenes in the GS mode are shown in Figure 3a. It is evident that in the presence of NaFl, (curve 1) as PAMPSA (curve 3) the synthesis proceeds at a lower potential than that in the presence of KPCF. In the PS mode, the synthesis charge grows significantly faster in the presence of NaFl (Figure 3b, curve 1) similar in PAMPSA (Figure 3b, curve 3). It can be assumed that NaFl creates favorable conditions for the formation of PEDOT chains and more effectively compensates for the positive charges on the PEDOT chains.

**Figure 4:**
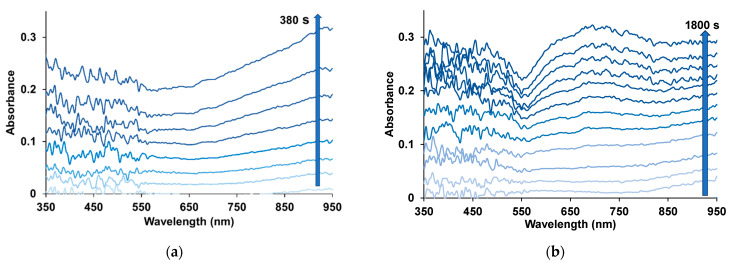
Electron absorption spectra of the PEDOT film formed on the working electrode during the polymerization of EDOT in the PS mode at the potential of 0.9 V in aqueous solutions of 0.0014 M NaFl (**a**) and 0.004 M KPCF (**b**). The optical path in the solution is 1.6 cm.

Simultaneously with the electrochemical parameters, electronic absorption spectra were recorded during the synthesis, the evolution of which is shown in Figure 4. It should be noted that the nature and dynamics of the spectral changes in the case of NaFl are characteristic of the synthesis of PEDOT in aqueous solutions [21] including in PAMPSA (Figure S3). A monotonic increase in the optical absorption is observed, most noticeable in the wavelength region above 600 nm and extending into the near-IR region. This indicates the formation of a PEDOT layer in the conducting form. In the presence of KPCF, an increase in the absorption in the 700 nm region is clearly visible, which corresponds to the polaronic form of PEDOT. Strong noise in the 350–550 nm region is associated with the subtraction of the intense absorption of fullerene solutions (Figure 1c), used as a background when recording the spectra.

**Figure 5:**
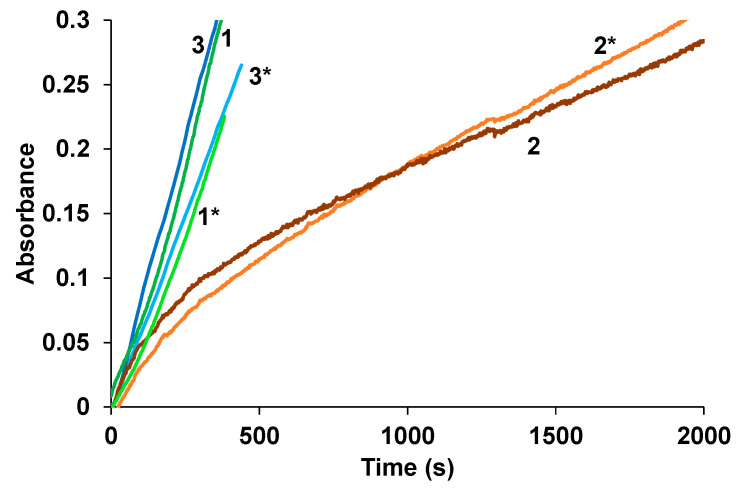
Time dependency of the optical absorption at 900 and 700 (*) nm during PS electrodeposition of PEDOT composite films in the presence of NaFl (1, 1*) and KPCF (2, 2*) and PAMPSA (3, 3*).

Figure 5 shows the dynamics of changes in the absorption of growing PEDOT films at characteristic wavelengths. It is evident that the polymerization of EDOT occurs at a higher rate in the presence of NaFl similar PAMPSA. Also, the absorption at 900 nm, characteristic of the bipolaron form of PEDOT, grows more actively in the case of NaFl (PAMPSA). In the presence of KPCF, the growth of the polaron form is more pronounced, and the growth of the bipolaron form begins to slow down toward the end of the synthesis. Such differences in kinetics of the syntheses may be associated with the tendency of KPCF to form aggregates of a large size in aqueous media, which may block the electrode surface and hinder the formation of PEDOT–KPCF films. This is also probably a result of steric hinderances caused by the asymmetric structure (a small part of the hydrophobic fullerene cage is covered with hydrophilic addends with negatively charged carboxyl groups) of KPCF and, accordingly, a worse ability to compensate for charges on the growing PEDOT chains.

### 3.2. Electron Absorption Spectroscopy of Composite Films in the UV-Visible and Near-IR Regions

**Figure 6:**
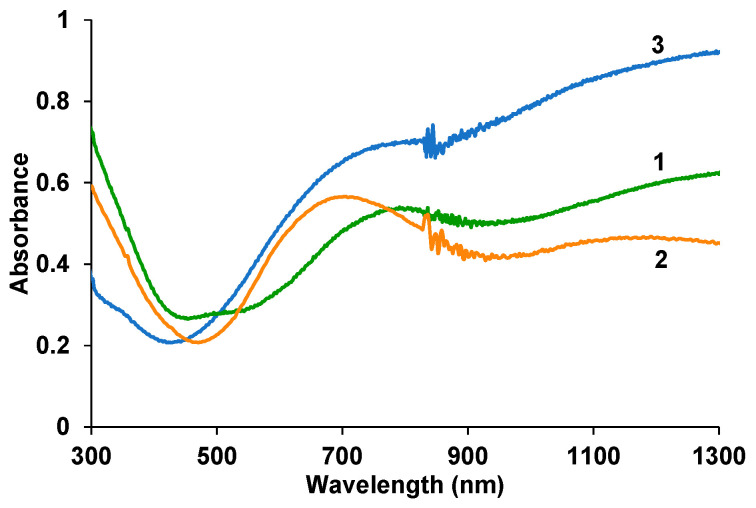
Electron absorption spectra in air of the PEDOT composite films electrodeposited in aqueous solutions of NaFl (1), KPCF (2), PAMPSA (3) at the charge density of 50 mC/cm^2^. The spectra were recorded in air.

Figure 6 shows the electron absorption spectra in the UV–visible and near-IR regions for PEDOT composite films with fullerenes. More intense absorption in the short-wavelength region is clearly visible compared to the PEDOT film obtained in the PAMPSA solution (curve 4), which corresponds to the absorption of both fullerenes (Figure 1c). Moreover, more intense absorption is observed in the case of NaFl, which indicates its higher content in the film. In contrast to the PEDOT–PAMPSA film (curve 3), the spectrum of which indicates that the polymer is in a highly conductive bipolaron state [22], for the composite films a pronounced absorption maximum is observed in the region of 700–800 nm, characteristic of PEDOT in the polaronic state [23]. Lower absorption in the near-IR region in the latter case is associated with a lower content of the bipolaron form. This trend is more pronounced for the PEDOT–KPCF composite (curve 2). In addition, in the spectrum of the PEDOT–KPCF composite, the absorption maximum of the polaron form is shifted to the short-wavelength region, which indicates shorter conjugation length [24].

### 3.3. Spectroelectrochemical Studies

**Figure 7:**
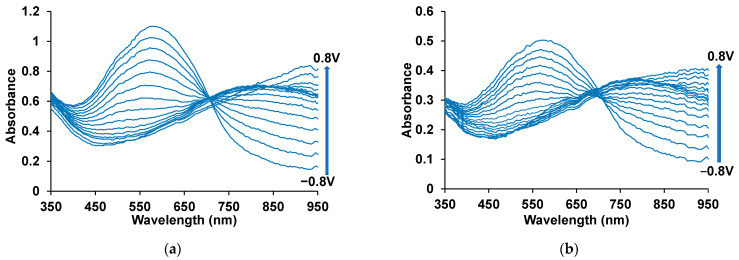
Electron absorption spectra of the composite films PEDOT–NaFl (**a**) and PEDOT–KPCF (**b**) measured at fixed potentials in 0.5 M NaClO4 aqueous solution.

The evolution of spectra depending on the fixed potentials are similar for both of the composite films. At low potentials (from −0.8 to −0.2 V), the band around 575 nm is observed, caused by π–π* transitions in the reduced form of PEDOT (Figure 7). As the potential increases (oxidation), the intensity of this band decreases, while within the potential range from −0.1 to 0.4 V, the absorption band around 800 nm is formed (the polaron form of oxidized PEDOT). Simultaneously, the absorption in the near IR region of the spectrum increases (bipolaron form) [25], which is most clearly expressed at the potentials from 0.5 to 0.8 V. The observed changes in the spectra during oxidation of the PEDOT composite film are characteristic of the PEDOT films studied in an aqueous medium [22,26]. However, the absorption maxima of the reduced and polaron forms of the composites are slightly shifted to the short-wavelength region compared to the PEDOT–PAMPSA film (Figure S4) (which has conventional spectroelectrochemical behavior) (Table 1), this shift being more pronounced for PEDOT–KPCF. This may be due to the formation of shorter PEDOT chains [24]. The steric hindrances for orientation of KPCF molecules with asymmetrically arranged carboxyl groups during the polymer formation lead to a worse ability to compensate positive charges on growing PEDOT chains. The same influence of steric hindrance on the conjugation length of PEDOT layers were observed in [26,27]. PEDOT layers were prepared in the presence of rigid-chain amid-containing polysulfonic acid and sulfonated poly(β-hydroxyethers) polyester with different content of sulfonic groups. The structure (rigidity of polyacid backbone and distribution of sulfonic groups) of the dopants creates a more strained structure for PEDOT chains and worse compensation of charges which results in the formation of chains with shorter conjugation.

By drawing tangents to the decay of the bands corresponding to the π-π* transition of the reduced form of PEDOT in composites of PEDOT with fullerenes (Figure 7), we determined the widths of the optical band gap for composites (Table 2).

### 3.4. Electrochemical Studies

**Figure 8:**
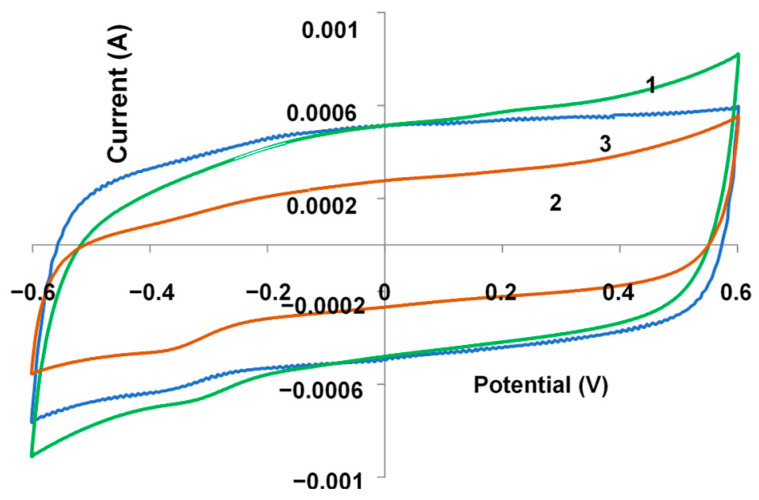
Cyclic voltammograms of PEDOT–NaFl (1), PEDOT–KPCF (2) and PEDOT-PAMPSA (3) films measured in 0.5 M NaClO_4_ aqueous solution at a scan rate of 100 mV/s. The films have equal electropolymerization charges.

Figure 8 shows the CVA of the composite films measured in 0.5 M NaClO_4_ solution in the range of potentials from −0.6 to 0.6 V (50 mV/s). Using them, the doping degree DD_CVA_ (Table 1) was calculated according to the relationship DD_CVA_ = 2Q_ox_/(Q_poly_–Q_ox_) [28,29], where Q_ox_ is the oxidation charge calculated by integrating the anodic waves of CVA measured in the same potential cycling range, and Q_poly_ is the polymerization charge spent on the electrosynthesis. In parallel with recording of SEM images we have performed energy dispersive X-ray (EDX) analysis of the samples (Figure S5). We calculated DD_EDX_ of composite films from these data. One can see that these values are in good correspondence with that determined from CVA (Table 1)_._ The doping degree of the PEDOT–KPCF composite is two times lower, which correlates with the lower absorption in the near-IR region of the spectrum. Apparently, in the presence of KPCF with unevenly distributed grafted benzene rings with carboxylate groups, some of these molecules are associated in low-mobile aggregates and there are steric hindrances for efficient electrostatic interactions of these groups with positively charged PEDOT chains. Moreover, the aggregates of KPCF may block some of the active growth sites on the electrode surface and thus hinder film formation. In the case of NaFl, the charge is uniformly distributed over the fullerene molecule. So, NaFl more efficiently compensates for the positive charges on the PEDOT chain, and a more optimal composite structure is formed. While comparing these values with those obtained for PEDOT-PAMPSA composite, one can notice that DD_CVA_ is much higher in the latter case and DD_EDX_ >> DD_CVA_. This situation is quite essential for PEDOT doped with polymeric acids [29] because growing PEDOT film may occlude excessive quantity of long macromolecules and not all sulfonic groups belonging to these macromolecules participate in the charge compensation (doping).

Figure S6 shows the CVA curves recorded in the non-aqueous medium. In the potential range of −0.6–0.2 V (Figure S6a) for the PEDOT–NaFl composite, a front of increasing anodic current of PEDOT oxidation is observed, from which it is possible to determine the HOMO level of PEDOT equal to −4.50 eV. A front of increasing cathodic current is also visible in the potential range of −1.2–1.0 V, which most likely characterizes the LUMO level of fullerene NaFl (E_LUMO_ = −4.05 eV) [30]. In the range of more negative potentials, a front of increasing cathodic current of PEDOT reduction is observed corresponding to E_LUMO(EC)_ = −2.97 eV. From the presented data, it is possible to calculate the width of the bandgap of the PEDOT–NaFl composite: E_g_ = 4.50 − 2.97 = 1.53 eV. Of note, this value is in good correspondence with that determined from the electron absorption spectra (Figure 7a) E_g(opt)_ = 1.55 eV.

For the PEDOT–KPCF composite, the front of the increasing anodic current of PEDOT oxidation is observed in the potential range of −0.7–0.5 V (Figure S6b), which is more cathodically than for PEDOT–NaFl and corresponds to E_HOMO_ = −4.33 eV. Taking into account approximately the same value of E_LUMO_ = −2.95 eV, the width of bandgap of the PEDOT–KPCF is E_g_ = 4.33 − 2.95 = 1.38 eV. This value is close to that determined from the electron absorption spectra (Figure 7b), E_g(opt)_ = 1.49 eV. Importantly, E_g_ for PEDOT–KPCF is lower than that for PEDOT–NaFl, demonstrating the same tendency as E_g(opt)_. Inside the bandgap in the range of potentials −1.4–1.0 V, one can see a front of increasing cathodic current, which characterizes the LUMO level of fullerene KPCF (E_LUMO_ = −4.05 eV). The energy characteristics of the substances are summarized in Table 2.

Relative positions of energy levels listed in Table 2 are graphically depicted in Figure S8.

Remark: for CV data and UPS data see in the Supplementary Materials in Figure S6 and Figure S7, respectively.

**Figure 9:**
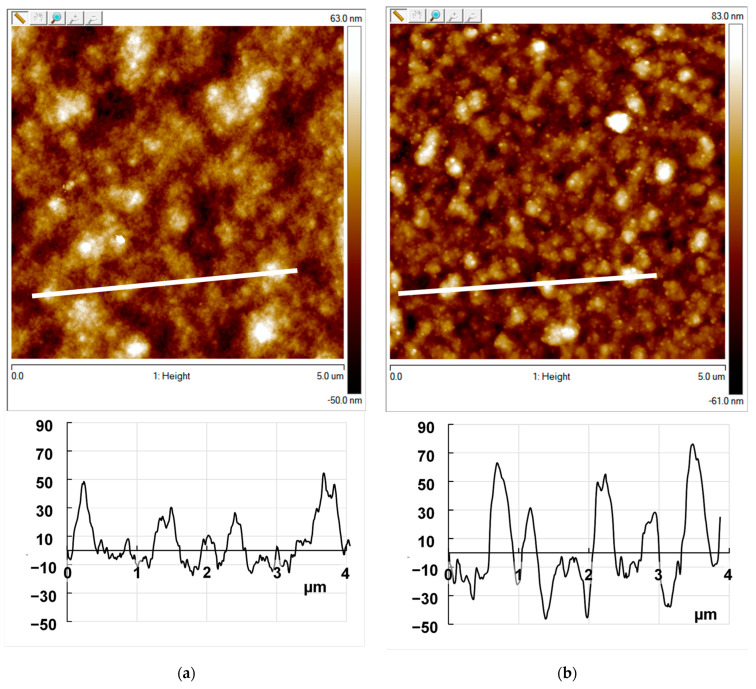

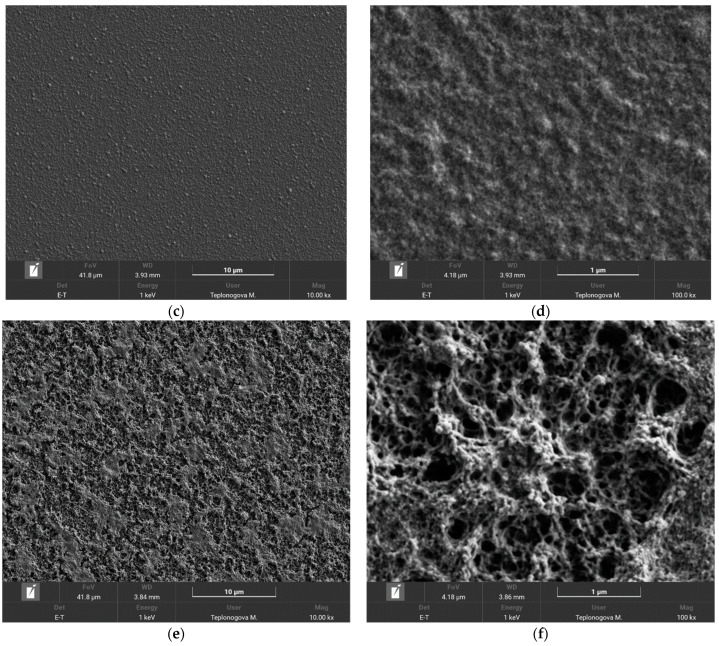
AFM images with profiles along white lines (**a**,**b**) and SEM (**c**–**f**) images of the surfaces of PEDOT composite films deposited onto FTO–electrodes with a PEDOT–PAMPSA sublayer in the presence of NaFl (**a**,**c**,**d**) and KPCF (**b**,**e**,**f**).

The bandgap reduction for the composite films with shorter chain length may seem a paradox. We can propose the only explanation - loose morphology of KPCF film (Figure 9f). From Table 1 it is clear that maxima of the absorption bands of the reduced forms of PEDOT-fullerene composites are blue-shifted compared to PEDOT-PAMPSA. This is normal situation for shorter chain length. At the same time loose, imperfect structure of the film contributes to increased dispersion of the energy levels, which manifests itself in broadening of absorption band. Since we determine the bandgap value from the intersection of a tangent to the red slope of absorption band with the axis of wavelengths, for broadened absorption band we obtain underestimated bandgap value. Similar situation is for CVA determination: loose structure of the film may facilitate appearing of the current onset at lower values of potential.

### 3.5. Morphology

The surface morphology of the PEDOT–NaFl and PEDOT–KPCF composite films was studied by AFM and SEM. From the AFM images one can see that PEDOT–NaFl (Figure 9a) has a filament-like structure that forms agglomerates with a lateral size 200–500 nm and height 30–50 nm and roughness 15 nm. PEDOT-PAMPSA film has similar structure with higher roughness 31.5 nm (Figure S9a,b). It should be noted that the roughness of PEDOT-NaFl film deposited on PEDOT-PAMPSA sublayer is lower than without sublayer (40–50 nm) [15]. It is known that the most defective morphology is formed on the stage of nucleation of a polymer phase on naked electrode. In our case the sublayer serves as nucleation centers resulting in formation of more homogeneous PEDOT-NaFl film. The surface of the PEDOT–KPCF film (Figure 9b) has a more globular structure with dense globules of a lateral size 250–350 nm, height 50–80 nm and a depression of depth about 50 nm. The roughness of PEDOT-KPCF film is 20 nm. The SEM overview images (Figure 9c,e) of the composites demonstrated uniform coatings on the electrodes without holes and cracks. The surface of the PEDOT–NaFl composite is more uniform and denser. At a higher magnification of the SEM image (Figure 9d), a granular surface of PEDOT–NaFl formed by compact and intergrown agglomerates was observed. The surface of PEDOT–KPCF is rougher with some hollows and hills (Figure 9f). One can see that their sizes (lateral and height/depth) are similar and correspond to the dimensions of aggregates 20 and 70 nm [20]. Some of them may be covered by PEDOT, which is forming the hills. The hollows may be formed as a result of washing out the substance from the surface during rinsing the films with water after the electropolymerization.

### 3.6. Near-IR Photoinduced Current

**Figure 10:**
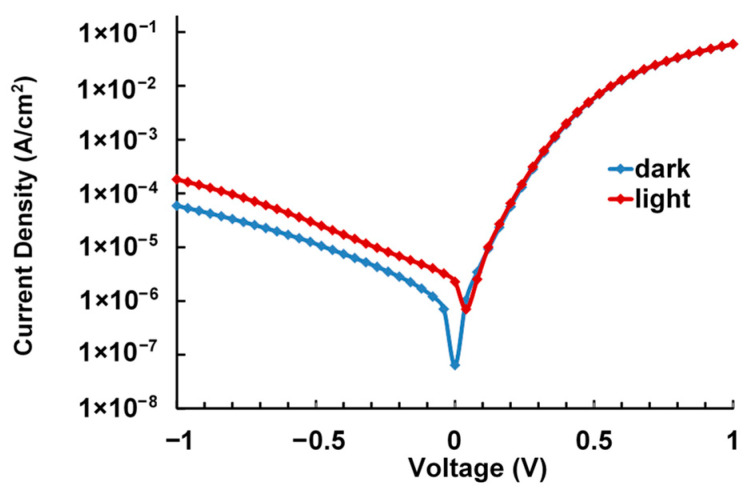
I–V characteristics of the PEDOT–NaFl based device in the dark (blue curve) and under near-IR illumination (brown curve) in the 700 nm–900 nm bandwidth at a power of 15 mW/cm^2^.

The photoconductivity was measured in devices of an ITO/sublayer/composite/C60/BCP/Al structure. The studied composites absorb radiation in the near-IR range due to PEDOT (Figure 6). The devices exhibited diode behavior. Typical current voltage characteristics are shown in Figure 10. Table 3 lists the near-IR photoinduced current and photosensitivity (photocurrent/incident power) in the PEDOT composites under irradiation in the 700 nm–900 nm bandwidth. In the fullerene-free PEDOT–PAMPSA layer, prepared for comparison, the change in conductivity under the near-IR irradiation was much smaller than that in the composites. In [31,32], the increase in current upon IR irradiation of a PEDOT:PSS film deposited by casting from a solution is explained to be caused by slight heating of the polymer film followed by increasing the charge carrier mobility. In the studied PEDOT–fullerene composites, we attribute the current induced by near-IR photoexcitation to an increase in the concentration of photogenerated charge carriers since fullerene can provide rapid exciton separation [33] by the transfer of photoexcited electrons from PEDOT to fullerene molecules. The transfer is energetically favorable according to the LUMO levels of the PEDOT donor and fullerene acceptor (Table 2). The higher response of PEDOT–NaFl compared to PEDOT–KPCF shows that the photoconductivity is associated with the influence of the fullerene structure on the degree of PEDOT doping and the morphology of the resulting layers. NaFl creates more favorable conditions for the formation of PEDOT chains and, as a consequence, a more uniform and denser layer of the composite with NaFl than with KPCF. The low current induced by near-IR radiation in the fullerene-free PEDOT–PAMPSA film indicates that the increase in current (charge carrier mobility) in the composites due to possible heating of the films makes a negligible contribution.

## 4. Conclusions

A comparative study of EDOT electropolymerization in aqueous solutions containing water-soluble fullerene derivatives with different ionogenic groups without adding supporting electrolytes was carried out for the first time. In this case, fullerenes were incorporated into the PEDOT films and acted as doping anions, compensating for the positive charges on the growing PEDOT chains.

It appeared that one of the composite layers (PEDOT–KPCF) can only be electrodeposited onto a thin PEDOT sublayer. Moreover, such sublayers usually served as hole-transporting and electron-blocking layers in various organic electronic devices.

It was demonstrated for the first time that the fullerene structure influences the rate and characteristics of PEDOT electrodeposition, degree of PEDOT doping and the morphology of the composite layers. The PEDOT–KPCF composite has a shorter conjugation length, a lower doping degree, and its surface is rougher with some hollows and hills. On the contrary, NaFl creates more favorable conditions for the formation of PEDOT chains and a more uniform and dense layer.

Photoconductivity of the composites of PEDOT with fullerene derivatives in the near IR spectral range has been demonstrated. The main result of the action of near-IR radiation on the composite films is the photogeneration of charge carriers due to the transfer of a photoexcited electron from PEDOT to an acceptor fullerene molecule.

## Figures and Tables

**Table 1:** Spectral characteristics and doping degrees of PEDOT with different counter ions.

Counterion	Maximum of the Reduced Form, nm	Maximum of the Polaronic Form, nm	DD_CVA_	DD_EDX_
NaFl	575	840	0.15	0.11
KPCF	573	776	0.08	0.06
PAMPSA	615	878	0.25	0.84

**Table 2:** Energy characteristics of the substances determined by different methods: E_g_—bandgap; E_f_—Fermi energy level; E_HOMO_—energy level of highest occupied molecular orbital; E_LUMO_—energy level of lowest unoccupied molecular orbital.

	E_g(opt)_, eV	From UPS Data		From CV Data			
		E_HOMO_, eV	E_f_, eV	E_LUMO_ PEDOT, eV	E_LUMO_ Fullerene, eV	E_HOMO_, eV	E_g_, eV
NaFl	2.78	−6.6	−4.8				
KPCF	2.63	−6.5	−4.9				
PEDOT–PAMPSA	1.57	−4.8	−4.8				
PEDOT–NaFl	1.55			−2.97	−4.05	−4.50	1.53
PEDOT–KPCF	1.49			−2.95	−4.05	−4.33	1.38

**Table 3:** Conductivity of composites under irradiation with the 700 nm–900 nm near-IR bandwidth. The incident power was 15 mW/cm^2^ and the reverse bias voltage was 0.04 V.

	PEDOT–NaFl	PEDOT–KPCF	PEDOT–PAMPSA
Photocurrent current, µA/cm^2^	2.5	0.54	0.02
Photosensitivity, μA/W	160	36	1.4

## Data Availability

Data are contained within the article and Supplementary Materials.

## Supplementary Materials

The following supporting information can be downloaded at: https://www.mdpi.com/article/10.3390/polym17010014/s1. Figure S1: Results of Zeta-potential measurements in 0.0005 M of NaFl (a) and KPCF (b). Electrolyte added: 10^−3^ M NaCl; Figure S2: Cyclic voltammogram measured during the PEDOT film deposition from an aqueous solution of 0.01 M EDOT and 0.02 M PAMPSA; Figure S3: Electron absorption spectra of the PEDOT film formed on the working electrode during the polymerization of EDOT in the PS mode at the potential of 0.9 V in aqueous solutions of 0.02 M PAMPSA; Figure S4: Eelectron absorption spectra of the PEDOT-PAMPSA film measured at fixed potentials in 0.5 M NaClO4 aqueous solution; Figure S5: Frame-averaged EDX spectra of PEDOT-KPCF (a) and PEDOT-NaFl (b) films electrodeposited on ITO transparent electrodes; Figure S6: Cyclic voltammetry of PEDOT-NaFl (a) and PEDOT-KPCF (b) electrodeposited into Pt-electrodes with PEDOT-PAMPSA sublayer. Electrolyte: 0.2 M Bu4NBF4 in AN. Scan rate: 20 mV/s; Figure S7: UPS spectra: secondary electron cutoff and valence band region of (a) NaFl, (b) KPCF and (c) PEDOT-PAMPSA films. The inset shows an enlarged view of spectrum on the semi-log plot to observe the shift of HOMO w. r. t. EF. Each energy value was determined with an experimental error of ±0.2 eV; Figure S8: HOMO and LUMO energy levels of NaFl, KPCF and PEDOT-PAMPSA obtained basing on UPS data and optical bandgap listed in Table 2; Figure S9: AFM image (a) and SEM (b) image of the surface of PEDOT-PAMPSA film deposited onto FTO-electrodes. Table S1. Frame-averaged content of the representative chemical elements in PEDOT-KPCF and PEDOT-NaFl films electrodeposited on ITO transparent electrodes.
