# Supplementary material for: Near-Infrared Responsive Composites of Poly-3,4-Ethylenedioxythiophene with Fullerene Derivatives
Source: Polymers (Basel). 2024 Dec 25;17(1):14. doi: 10.3390/polym17010014 (PMC11723435; doi:10.3390/polym17010014)
Supplement: Supplementary file 1 [file polymers-17-00014-s001.zip › polymers-3345988-supplementary.pdf]

Supplementary materials

Near-Infrared Responsive Composites of Poly-3,4-ethylenedioxythiophene with Fullerene Derivatives

Oxana Gribkova, Varvara Kabanova, Ildar Sayarov, Alexander Nekrasov and Alexey Tameev*

Zeta-potential measurements.

The ζ-potential of the fullerenes solutions at the concentration of 0.0005M was measured by means of Zetasizer Nano ZS (Malvern) analyzer.

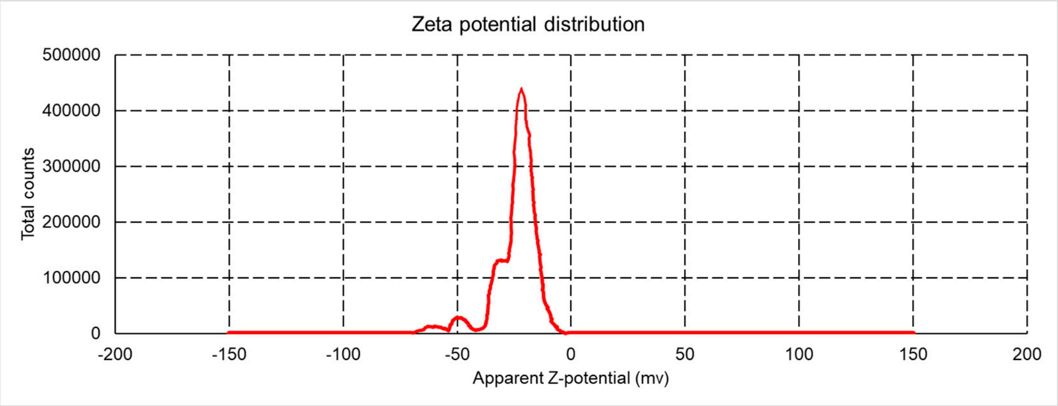

(a)

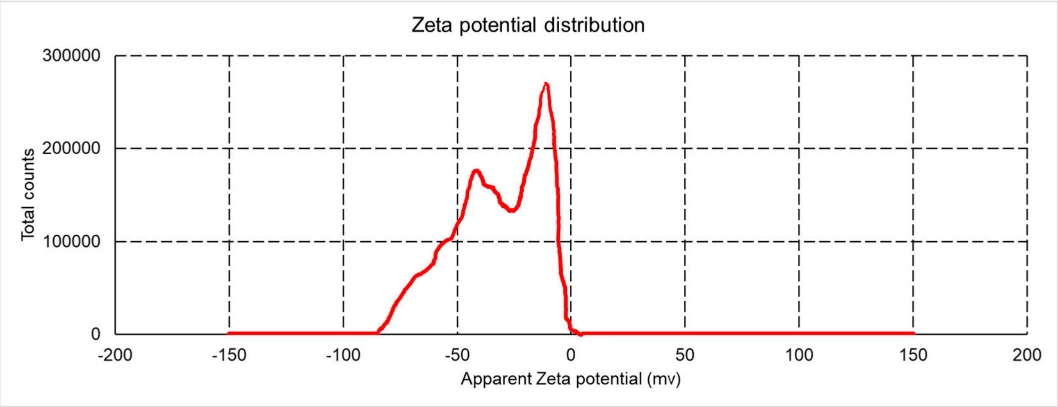

(b)

Figure S1. Results of Zeta-potential measurements in 0.0005M of NaFl (a) and KPCF (b). Electrolyte added: 10^{-3}M NaCl.

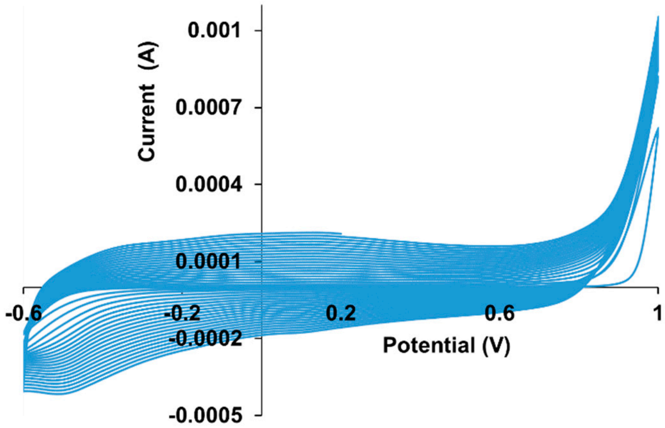

Figure S2. Cyclic voltammogram measured during the PEDOT film deposition from an aqueous solution of 0.01 M EDOT and 0.02 M PAMPSA.

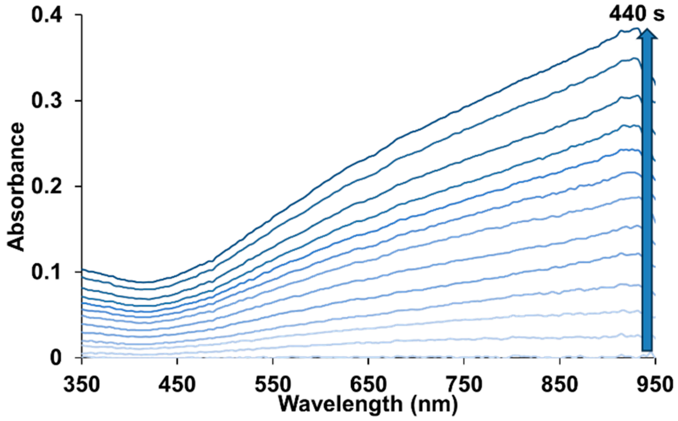

Figure S3. Electron absorption spectra of the PEDOT film formed on the working electrode during the polymerization of EDOT in the PS mode at the potential of 0.9 V in aqueous solutions of 0.02 M PAMPSA.

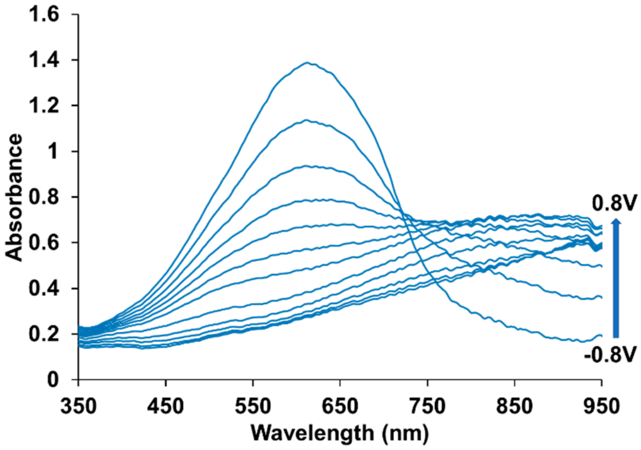

Figure S4. Eelectron absorption spectra of the PEDOT-PAMPSA film measured at fixed potentials in 0.5 M $NaClO_4$ aqueous solution

EDX mapping

In parallel with recording of SEM images we have performed EDX mapping of the samples for the frames of ca. 15 × 25 μm size (Fig. S5). For these experiments we have used PEDOT-KPCF and PEDOT-NaFl samples that were prepared for photoelectrical measurements, i.e., the films applied onto ITO transparent electrodes. The EDX mapping showed the presence of Si, In and Sn, which are the components of the optically transparent conductive substrate: glass (SiO_2) and ITO (In_2O_3 and SnO_2). Therefore, the oxygen atoms contained in these oxides should be excluded from the calculation of the EDOT-fullerene ratio (Table S1, sum column). Content of oxygen in the fullerene derivatives O_{calc} (fullerene) was calculated by subtraction of the value from the sum column from the total measured content O_{exp}. Taking into account, that KPCF molecule contains 5 COOK groups (Figure 1a and ref. [16] in the main text) and each -COOK group contains 2 oxygen atoms the content ratio COOK/EDOT = 0.058. Taking into account, that NaFl molecule 30 OH groups (Figure 1c and ref. [16] in the main text) the content ratio OH/EDOT = 0.104.

Similar value for PEDOT-PAMPSA composite films SO_3/EDOT = 0.84.

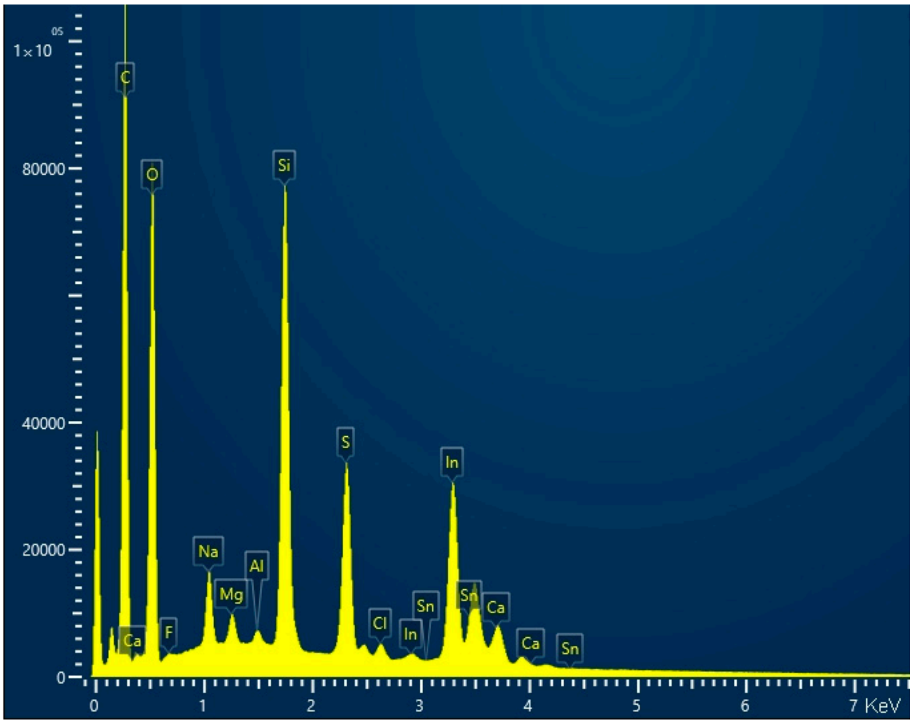

(a)

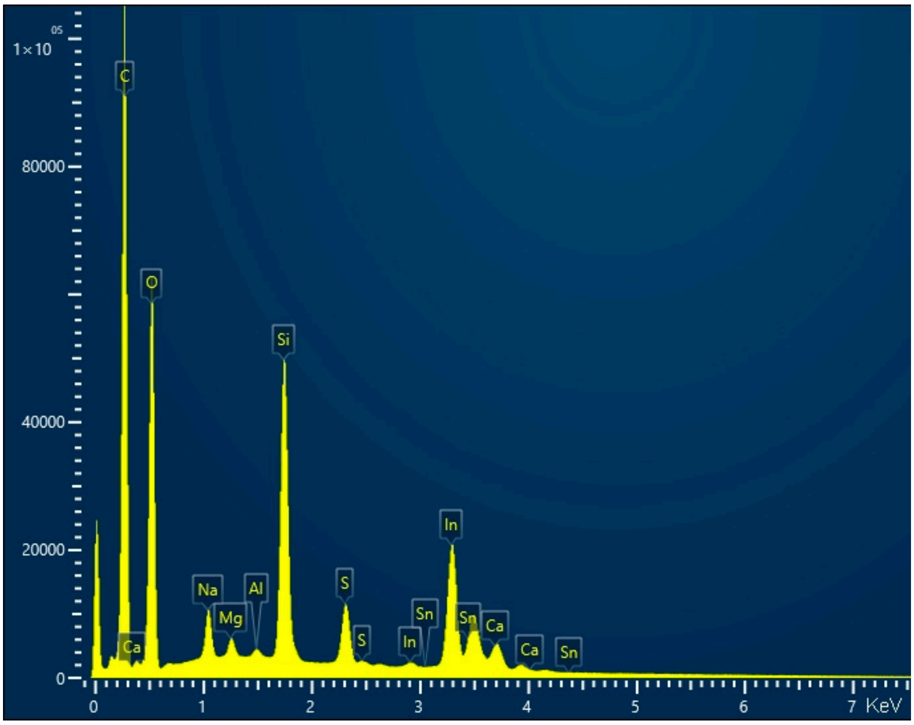

(b)

Figure S5. Frame-averaged EDX spectra of PEDOT-KPCF (a) and PEDOT-NaFl (b) films electrodeposited on ITO transparent electrodes

Table S1. Frame-averaged content of the representative chemical elements in PEDOT-KPCF and PEDOT-NaFl films electrodeposited on ITO transparent electrodes

	Frame-averaged content / atom. %									
	S_{exp}	O_{exp}	Si_{exp}	In_{exp}	Sn_{exp}	O_{calc}				O_{calc} (fullerene)
						SiO_2	In_20_3	SnO_2	sum	
PEDOT- KPCF	8.59	56.16	16.16	11.25	1.00	32.32	16.88	2.00	51.20	4.97
PEDOT-NaFl	1.78	28.15	6.99	5.23	0.39	13.98	7.85	0.78	22.61	5.55

Determination of HOMO and LUMO energy levels by cyclic voltammetry.

To determine the HOMO and LUMO levels of the composite films were electrodeposited on Pt electrodes of 5×5 mm size. The electropolymerization solutions were the same as described in the main text of the manuscript. First a PEDOT-PAMPSA sublayer was electrodeposited it galvanostatic (GS) mode at 0.05 mA/cm^2 until 7 mQ/cm^2 charge was reached. Then PEDOT-fullerene composite films were deposited on the sublayer in GS-mode at 0.05 mA/cm^2 until 100 mQ/cm^2 charge was reached. Cyclic voltammograms of the composite films were recorded in an inert argon atmosphere in a three-electrode cell with separated volumes for the working and auxiliary (Pt) electrodes, as well as an Ag/$AgNO_3$ pseudo-reference electrode (Ag wire in an electrolyte solution with the addition of $AgNO_3$). A 0.2 M solution of tetrabutylammonium tetrafluoroborate (Bu_4NBF_4) in deoxygenated acetonitrile (AN) was used as the electrolyte.

The values of potential corresponding to HOMO and LUMO energy levels were determined by plotting tangents to the oxidation (HOMO) or reduction (LUMO) current fronts until they intersected the axis of potential. To recalculate the values of potential to the scale of energies the pseudo-reference electrode was calibrated relative to the ferrocene/ferricinium couple, the energy level of which in an acetonitrile solution is 4.988 eV. For this experiment, a PEDOT-NaFl and PEDOT-KPCF films were electrodeposited in the GS mode onto the Pt electrode covered with PEDOT-PAMPSA sublayer and then dried in vacuum.

If one compares the CVs for PEDOT-NaFl and PEDOT-KPCF (Figure S6) one can notice much lower redox charge in the range of potentials from -0.6 to -0.2V in the latter case. This correlates well with the lower doping degree of PEDOT-KPCF measured on FTO electrodes (Table 1 in the main text).

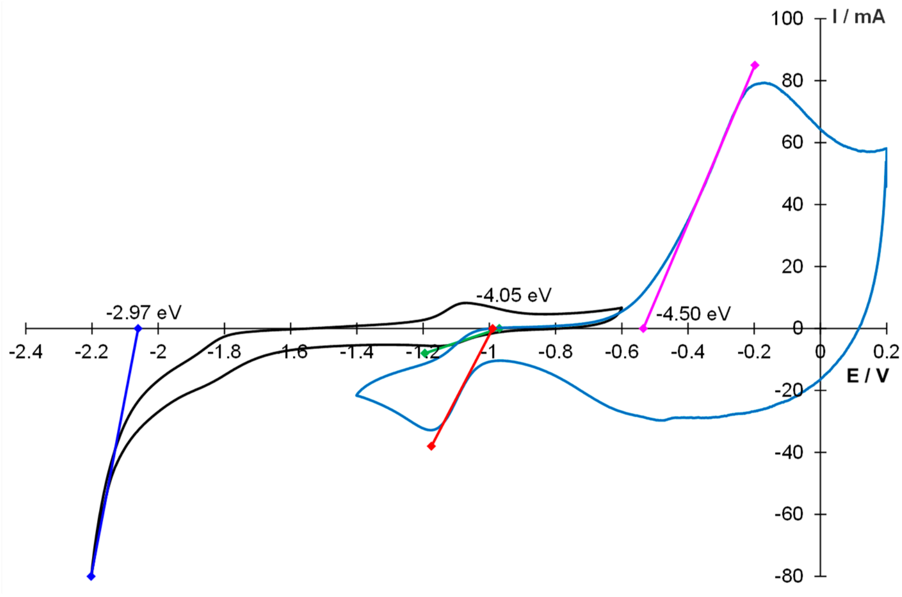

(a)

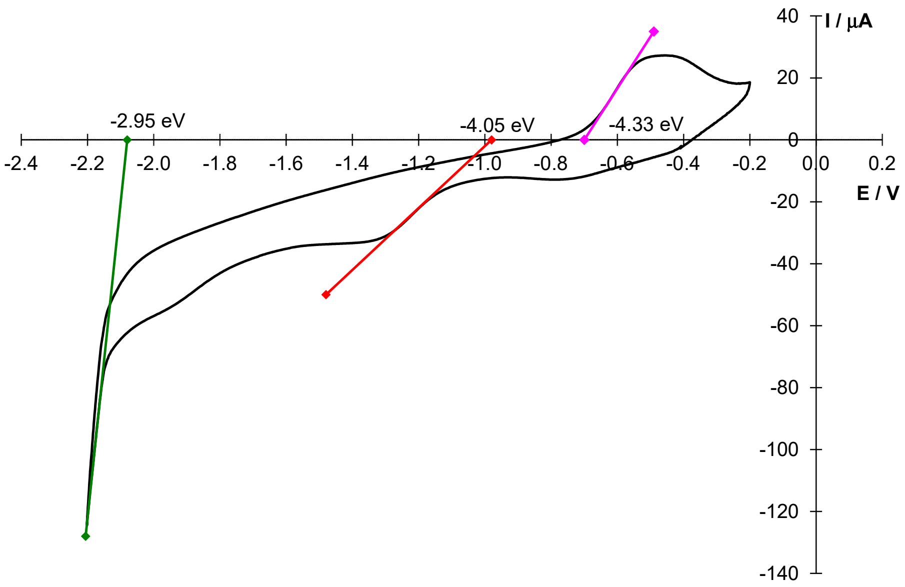

(b)

Figure S6. Cyclic voltammetry of PEDOT-NaFl (a) and PEDOT-KPCF (b) electrodeposited into Pt-electrodes with PEDOT-PAMPSA sublayer. Electrolyte: 0.2 M Bu_4NBF_4 in AN. Scan rate: 20 mV/s.

Comparison of CVs for PEDOT-NaFl composite film on the sublayer and on pure Pt (ref. [15] in the main text) reveals only slight difference in the amplitude of current. Most possibly this is due to incomparable thickness of the sublayer and the composite film, as well as due to the fact, that very thin PEDOT-PAMPSA layer is in contact with Pt, which extracts electros from this sublayer thus increasing its hole conductivity.

UPS spectra

(a)

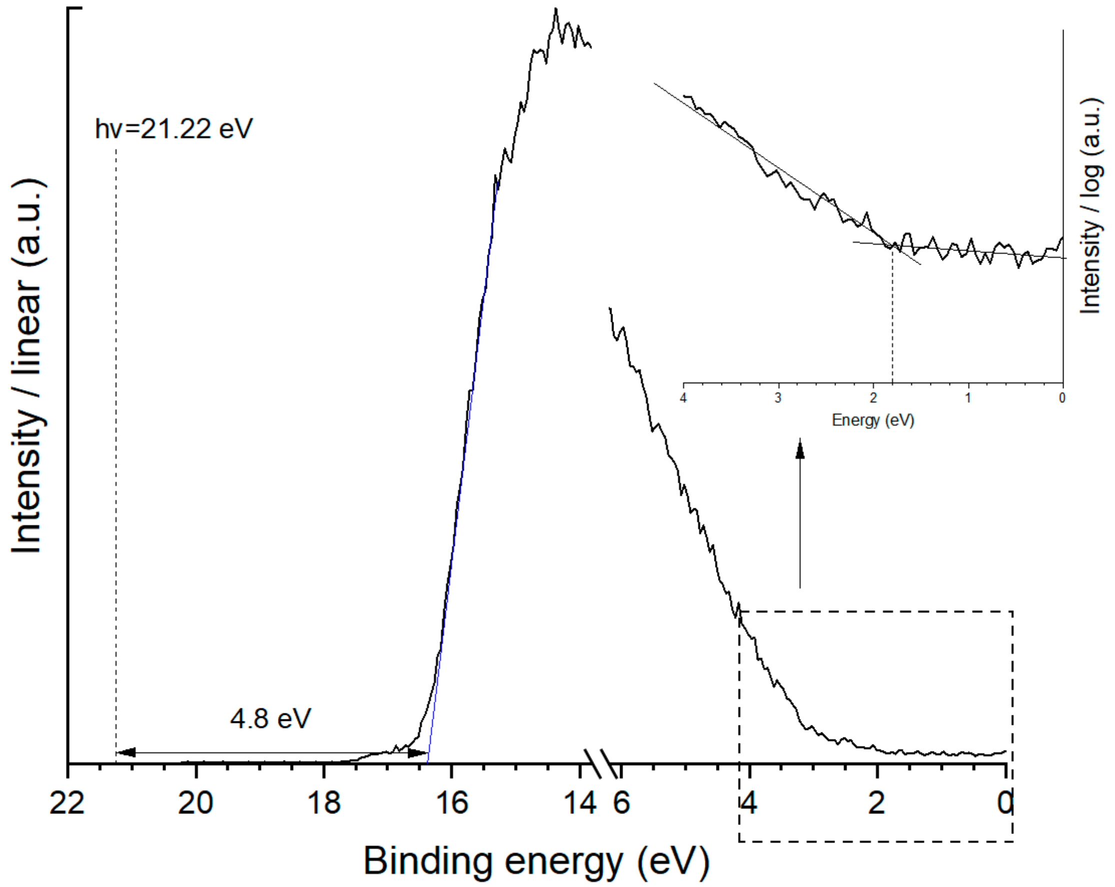

(b)

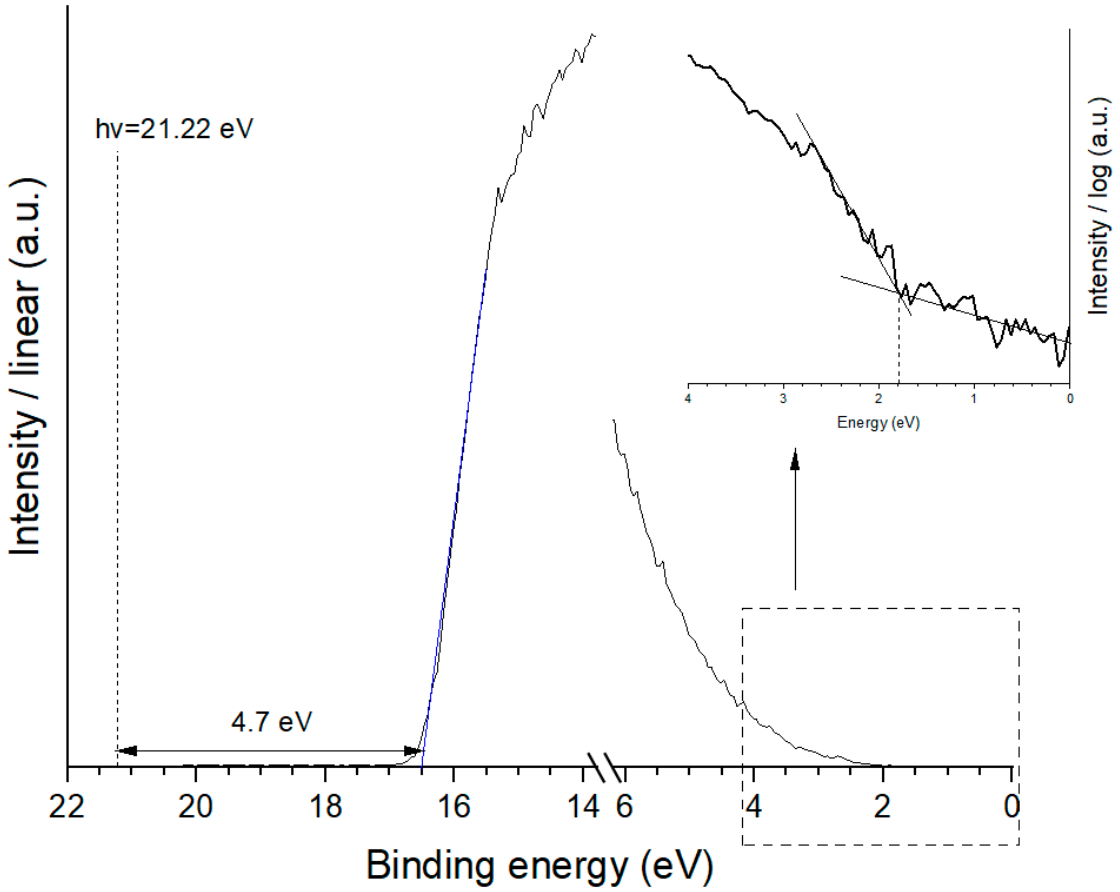

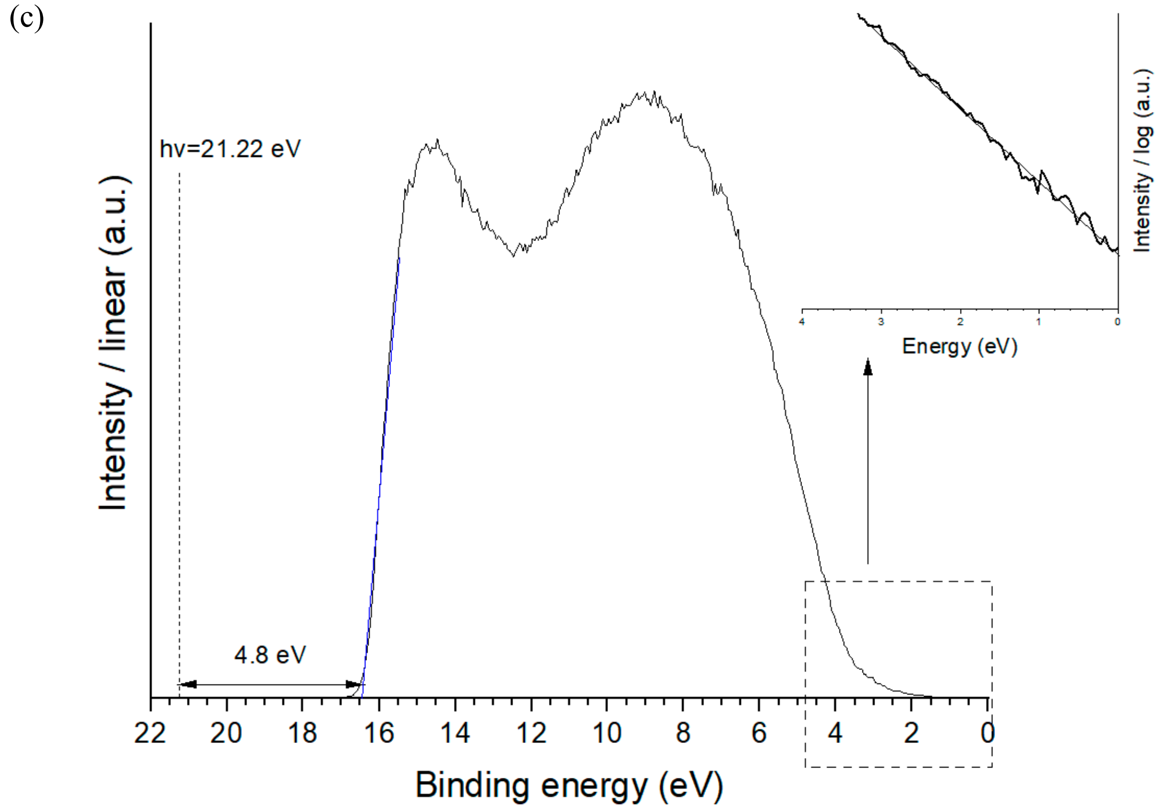

Figure S7. UPS spectra: secondary electron cutoff and valence band region of (a) NaFl, (b) KPCF and (c) PEDOT-PAMPSA films. The inset shows an enlarged view of spectrum on the semi-log plot to observe the shift of HOMO w. r. t. E_F. Each energy value was determined with an experimental error of ±0.2 eV.

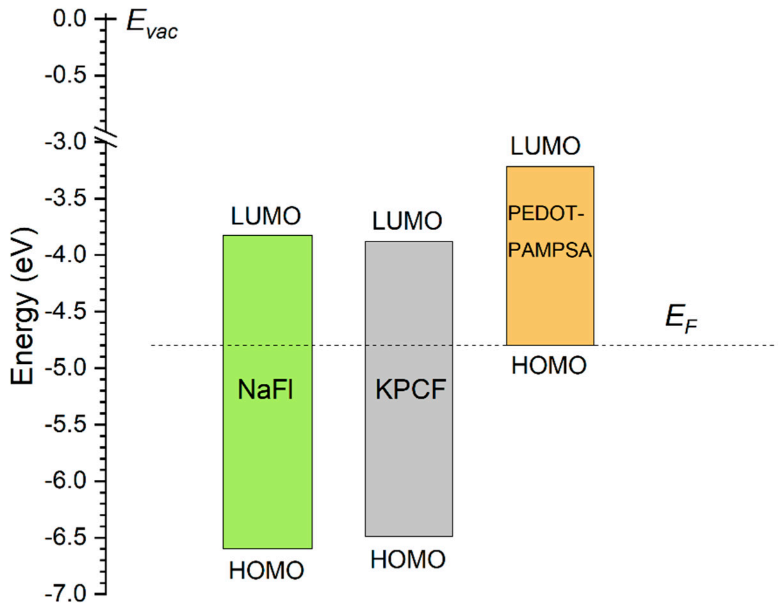

Figure S8. HOMO and LUMO energy levels of NaFl, KPCF and PEDOT-PAMPSA obtained basing on UPS data and optical bandgap listed in Table 2.

AFM and SEM images

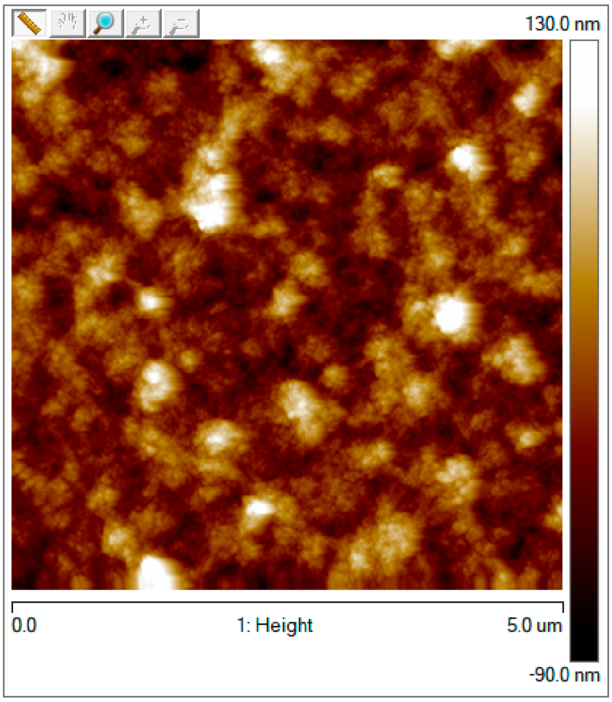

(a)

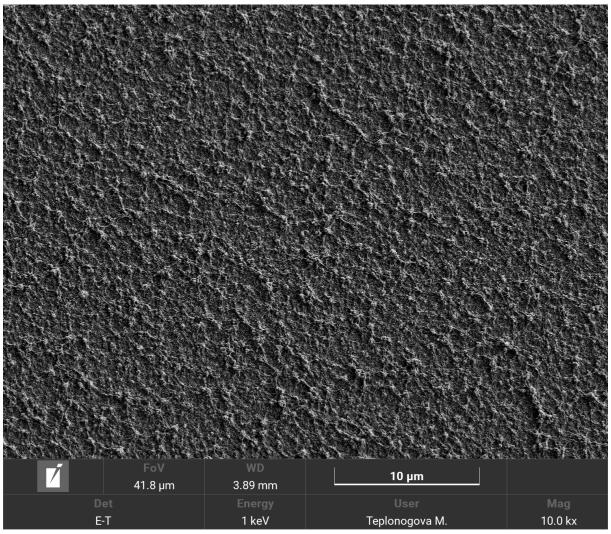

(b)

Figure S9. AFM image (a) and SEM (b) image of the surface of PEDOT-PAMPSA film deposited onto FTO-electrodes.
